# Changes in root microbiome during wheat evolution

**DOI:** 10.1186/s12866-022-02467-4

**Published:** 2022-02-26

**Authors:** Somayeh Gholizadeh, Seyed Abolghasem Mohammadi, Ghasem Hosseini Salekdeh

**Affiliations:** 1 Department of Plant Breeding and Biotechnology, Faculty of Agriculture, University of Tabriz, Tabriz, Iran; 2 Center of Excellence in Cereal Molecular Breeding, University of Tabriz, Tabriz, Iran; 3 Department of Molecular Sciences, Macquarie University, Sydney, NSW Australia; 4 Department of Systems Biology, Agricultural Biotechnology Research Institute of Iran (ABRII), Agricultural Research Education and Extension Organization (AREEO), Karaj, Iran

**Keywords:** Wheat species, Domestication, Breeding, Genotype, Developmental stage, Differential abundance, Microbiome, Rhizosphere

## Abstract

**Background:**

Although coevolutionary signatures of host-microbe interactions are considered to engineer the healthy microbiome of humans, little is known about the changes in root-microbiome during plant evolution. To understand how the composition of the wheat and its ancestral species microbiome have changed over the evolutionary processes, we performed a 16S rRNA metagenomic analysis on rhizobacterial communities associated with a phylogenetic framework of four *Triticum* species *T. urartu*, *T. turgidum*, *T. durum*, and *T. aestivum* along with their ancestral species *Aegilops speltoides*, and *Ae. tauschii* during vegetative and reproductive stages.

**Results:**

In this study, we illustrated that the genome contents of wild species *Aegilops speltoides* and *Ae. tauschii* can be significant factors determining the composition of root-associated bacterial communities in domesticated bread wheat. Although it was found that domestication and modern breeding practices might have had a significant impact on microbiome-plant interactions especially at the reproductive stage, we observed an extensive and selective control by wheat genotypes on associated rhizobacterial communities at the same time. Our data also showed a strong genotypic variation within species of *T. aestivum* and *Ae. tauschii*, suggesting potential breeding targets for plants surveyed.

**Conclusions:**

This study performed with different genotypes of Triticum and Aegilops species is the first study showing that the genome contents of *Ae*. *speltoides* and *Ae. tauschii* along with domestication-related changes can be significant factors determining the composition of root-associated bacterial communities in bread wheat. It is also indirect evidence that shows a very extensive range of host traits and genes are probably involved in host-microbe interactions. Therefore, understanding the wheat root-associated microbiome needs to take into consideration of its polygenetic mosaic nature.

**Supplementary Information:**

The online version contains supplementary material available at 10.1186/s12866-022-02467-4.

## Background

The rhizosphere, a narrow soil zone around the roots, is home to an immense number of microorganisms [1] which can have profound advantages on plant nutrient uptake [2], growth [3], drought tolerance [4], life cycle phenology [5], and disease resistance [2]. Photosynthesis-derived organic compounds called root exudates are key factors of rhizosphere microbiome assemblage [6]. The composition of root exudate profiles can change in different plant species, genotypes, and developmental stages [7].

Several studies have demonstrated the species-specific effects of plants on the composition and relative abundance of bacterial populations in the rhizosphere of crops [8, 9]. Understanding how the composition of the host species microbiome has changed over the evolutionary processes requires the inclusion of the microbiomes of phylogenetic outgroups (i.e., wild and close species) into analyses of plant microbiomes. In several studies, a correlation between host phylogenetic distances and the clustering of rhizosphere microbiome was reported for *Poaceae* species [10], *Brassica napus* [11], different species from monocots and dicots [12], but not for distant relatives of Arabidopsis [13]. In addition, there are some pieces of evidence for coevolution between cereals and their seed microbiome as a consequence of domestication [14, 15]. Domestication may also change root exudation, root architecture, and plant defense mechanisms [16], therefore differences in rhizosphere microbiome would be predicted due to plant domestication. Distinct rhizobacterial communities were observed between domesticated plants and their wild ancestors in barley [17], maize [18], wheat [19], Agave [20], and common bean [21] compared with their respective wild ancestors.

Not only the plant species and their evolutionary processes but also the host genotypes can influence the composition of the rhizosphere microbiome. Initial studies conducted using the model plant *Arabidopsis thaliana* [22, 23] and more recent studies on rice [24] and a wild perennial plant of *Brassicaceae* [25] revealed that the host genetic materials shape root microbiome profiles. The extent of plant genetic control over root bacterial communities is of interest to both evolutionary biologists and crop breeders because the heritability of the root microbiome declares whether microbial communities can evolve in response to selection on host plants [25].

Crop breeding programs are typically performed in monoculture systems under fertile conditions and in the absence of soil-borne pathogens and abiotic stress, thus keeping the contribution of the beneficial rhizosphere microbiome to plant growth and health to a minimum. In such conditions, it has been assumed that modern plant breeding may have been selected against plant traits that are critical for the recruitment and support of beneficial microorganisms [26]. For instance, in maize, the local landraces were significantly more colonized by mycorrhizal fungi and obtained more phosphorus under low and medium phosphate concentrations than did the modern maize hybrid [27]. Similar results were observed for fungal and bacterial populations colonized the root system of ancient versus modern lines [26, 28], and tall compared to semi-dwarf cultivars [29] of wheat.

**Fig. 1:**
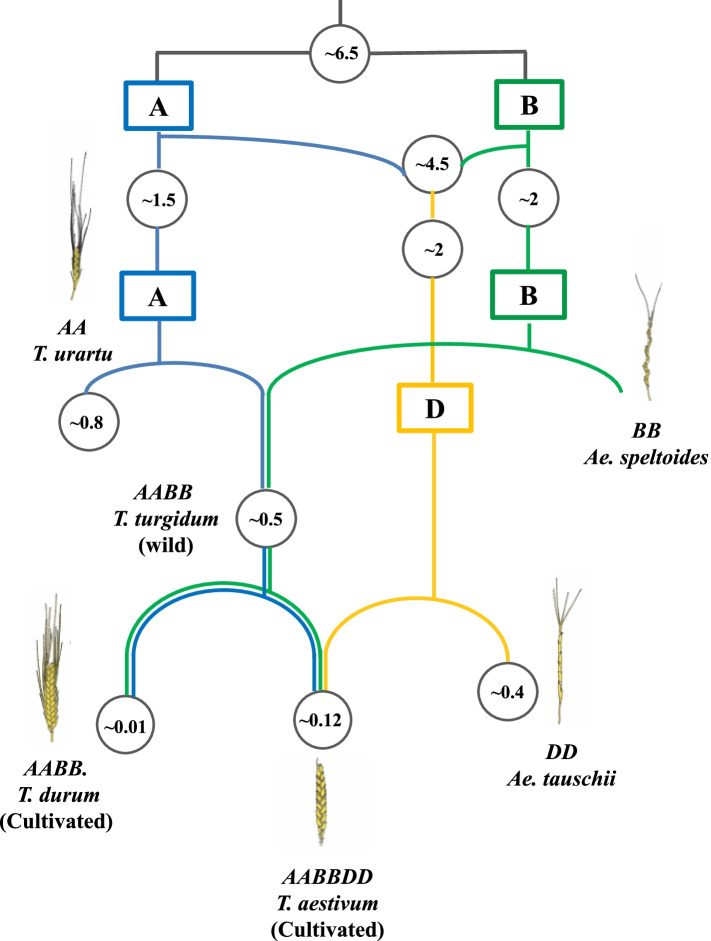
Model of the phylogenetic history of hexaploid bread wheat (*Triticum aestivum*; AABBDD) based on Marcussen and coworkers [33]. Approximate dates for divergence and different hybridization events are given in white circles in units of million years ago

Temporal scales are also of crucial importance in the rhizosphere as the amount and composition of rhizodeposits can also vary in time during the growth and root development of a single plant species [1]. For example, distinct rhizobacterial communities were associated with different developmental stages of Arabidopsis [6], *Arabis alpine* [30], wheat [8], and common bean [31]. *Triticum aestivum* (bread wheat, hexaploid genome; AABBDD) and its relatives offer an ideal system for studying evolutionary theory [32]. As summarized in Fig. 1, two bread wheat subgenomes A (*T. urartu*; AA) and B (*Aegilops speltoides*; BB) diverged from a common ancestor approximately 7 million years ago, and these genomes gave rise to the D genome through homoploid hybrid speciation one to two million years later [33]. The initial allopolyploidization event comprised the A and B genome donors, resulting in the existent tetraploid wild emmer wheat (*T. turgidum*; AABB). During agricultural development, *T. turgidum* was domesticated and their cultivated forms emerged [34]. This species subsequently hybridized with the D genome donor to develop modern hexaploid bread wheat (Fig. 1).

Since its origin in the Fertile Crescent 10,000 years ago, wheat has undergone dramatic phenotypic changes concerning cultivation conditions and human needs. It has also been speculated that these processes have also influenced belowground traits, including root architecture [35, 36] and root exudate composition [37, 38], which in turn may modify rhizosphere microbial communities in ways that are still poorly understood.

In this paper, we examine the influence of wheat species, domestication, genotype, and modern breeding on plant-microbiome interactions at two developmental stages, including seedling (vegetative) and flowering (reproductive) times. We asked four questions: (a) How do different developmental stages affect the wheat rhizosphere microbiome? (b) Do host evolutionary differences shape the structure of the bacterial communities of wheat species? (c) How do host genetic diversities affect the wheat root-soil interface? and (d) Whether the processes of domestication and modern breeding have left a human footprint of selection on the wheat-associated microbiome?

## Methods

### Soil collection

The planting medium used in the current study was a combination of live and autoclaved soil mix. Surface soils were collected from the top 20 cm depth of the wheat fields and grassland ecosystems (Supplementary Table 1), transported to the lab at room temperature, and stored in a cold room (4–5 °C) until further use. All types of soil were selected for this study in the north, north-west and west regions of Iran (Supplementary Table 1). These regions are located in the center of origin of wild wheat relatives and local genotypes. All grassland sites were free of pesticide application and heavy human traffic, and wheat field sites also were under fallow management and were not exposed to agrochemicals for more than 10 years. The visible weeds, twigs, worms, insects, and debris were removed and the soils were then crushed with an aluminum mallet to a fine consistency and sifted through a sterile 2-mm sieve. The sieved soils from different sites then combined and represented live soil used as a microbiome seed bank. Live soil was subsequently mixed with sterile potting soil including Peat: Vermiculite (1:1 v/v) autoclaved for 9 h at 121 °C, resulting in nutrient-depleted soil conditions with low levels of nitrate and phosphorus (Supplementary Table 2) and used for all genotypes.

### Sample preparation

We collected seeds of 31 genotypes of different wheat species shown in Supplementary Table 3 and Supplementary Table 4. The genotypes of *Ae. speltoides* were kindly provided by Seed and Plant Improvement Institute (SPII), Karaj, Iran. Wheat cultivated genotypes and other wild accessions were also donated by the Center of Excellence in Cereal Molecular Breeding, University of Tabriz, Tabriz, Iran. Our germplasms included seven genotypes of *T. aestivum*, five genotypes of *T. durum*, two accessions of *T. turgidum*, six accessions of *T. urartu*, five accessions of *Ae. tauschii*, and six accessions of *Ae. speltoides*. Seeds were surface-sterilized by treatment for 2 min in 70% ethanol with 0.1% Triton-X100 for 2 min, followed by 10 min in 2.5% bleach with 0.1% Triton-X100 and washed with sterile distilled water by taking in turns for 3–5 times, followed by three washes in sterile distilled water. Seeds were placed on 0.5% agar containing half-strength Murashige & Skoog (MS) vitamins and 1% sucrose [22]. They were germinated at 18 °C under 18 h of light for 2 weeks. Seed coat sterility was confirmed by a lack of visible contamination on MS plates during germination. Two-week-old healthy seedlings were aseptically transplanted from MS plates to sterile pots (14*10.5*14 Cm) filled with mixed soil, with two seedlings per pot. Some pots were designated bulk soil with no plant. For all pots, including planted and bulk soil controls, nitrogen was applied as 200 ml ammonium nitrate at 0.5 g/L with the first water. After that, all pots were always watered with a shower of distilled water (non-sterile) as an accessible proxy for rainwater that avoids chlorine and other tap water additives. Pots were spatially randomized and placed in growth chambers providing short days of 8 h light (80000–100,000 lx) and 16 h dark at 14 °C and relative humidity of 40%. After 4 weeks, the temperature was weekly decreased 3 °C to reach 5 °C to take place the vernalization process for 6 weeks [39]. The use of low temperatures and short days was to help synchronize development time between wild and cultivated genotypes and to facilitate root growth and flowering time [22]. After a 6-week-period of vernalization, the temperature weekly increased 3–4 °C to reach 18 °C to achieve the optimum temperature for a more synchronized growth and development. Plants were harvested at the seedling growth stage (vegetative stage) when there were 5–7 leaves on each seedling. Then, the remaining plants and bulk soils experienced 16-h days at 18 °C in the growth chamber to promote a more synchronized flowering stage. The flowering harvest (reproductive stage) started when One-thirds of all genotypes showed anthesis; this occurred approximately 7 weeks after the first sampling time. We considered the flowering time for every genotype when anthesis began in the central part of the spike for every 3 replications of the genotype.

### Sample collection

Samples were collected over a 7-day and 14-day period for vegetative and reproductive stages, respectively. The soil and plant were removed from each pot and the roots were separated from the soil. We avoided collecting any roots that were at the interface of the pot and the soil to avoid false environments. The excess soil was manually shaken from the roots, leaving approximately 1 mm of soil still attached to the roots. We placed the roots in a sterile flask with 50 ml of sterile Phosphate Buffered Saline (PBS) solution to separate the remaining soil (rhizosphere) from the roots. The soil that was cleaned from the roots was poured into a 2 ml tube, which was then spun at 10000 g for 30 s to form tight pellets, from which all supernatant was removed, snap-frozen, lyophilized, and stored as the rhizosphere at − 20 °C until DNA extraction. Bulk soil samples were collected from unplanted pots approximately 2 in. below the soil surface during sampling days and kept as same as the rhizosphere. We attempt to represent all genotypes and at least one bulk soil during harvest days to avoid potential confounding harvesting artifacts with genotype effects.

### DNA extraction, amplification, and sequencing

To extract DNA, the rhizosphere and bulk samples were suspended in a lysis buffer and microbial cells were mechanically lysed through SDS and bead beating. For all bulk soil and rhizosphere data, bead beating and purification were performed with the E.Z.N.A soil DNA extraction kit (Omega Bio-Tek, Inc., Norcross, GA, USA) according to the manufacturer’s instructions. Metagenomic profiling of the samples was performed by sequencing the V3-V4 region of the 16S rRNA gene. In brief, we used 341F and 785R primer sets (341 F, 5′-CCTACGGGNBGCASCAG-3′ and 785 R, 5′-GAC TACNVGGGTATCTAATCC-3′) and a 2-step PCR, where the first PCR amplified the V3-V4 region, and the second PCR extended the amplicons with adapters and barcodes. PCR reactions were made using Thermo Scientific™ Taq DNA Polymerase and Thermo Scientific™ dNTP set. Each reaction was done in a volume of 25 μL using 12.7 μL H2O PCR grade, 2.5 μL PCR Buffer, 1 μL forward primer (10 μM), 1 μL reverse primer (10 μM), 1 μL sample DNA (~ 50–100 ngμl^− 1^), 2 μL MgCl2, 2 μL dNTPs, BSA (0.1 μgml^− 1^) 2.5 μL and 0.3 μL polymerase. This PCR reaction was performed in triplicate along with negative control to reveal contamination. The PCR program used was 94 °C for 5 min followed by 25 cycles each of 94 °C for 20 s, 55 °C for 30 s and 72 °C for 40 s, followed by 72 °C for 10 min. The reactions were run on a 1% agarose gel to ensure the amplification was successful. We first verified that the no-template control did not contain DNA via gel electrophoresis and then pooled the three replicate PCR products and purified them with PureLink™PCR Purification Kit (Invitrogen, Carlsbad, CA, USA) to clean the 16S V3-V4 amplicon from free primers and primer-dimer species. Then the barcoded PCR reactions with 2 μl of the purified PCR product were performed in triplicate as same as the first PCR except for primer sets containing adapters and a barcode attached for annealing to the Illumina flow cell and 8-cycle annealing. Next, three replicates of index PCR products from each sample were cleaned up using Gene JET PCR Purification Kit (Thermo Fisher Scientific, Waltham, MA, USA) on agarose gel 2%, pooled, and qualified with PicoGreen (Invitrogen). PCR products from 207 libraries were then combined in equimolar ratios into a master DNA pool and sequenced by Macrogen (Seoul, South Korea) for 250 × 250 paired-end, dual index sequencing on an Illumina MiSeq instrument.

### Data processing

The sequences obtained from the MiSeq run were demultiplexed based on the barcode sequences using a custom Perl script based upon exact matching. After we assembled read1 and read2 with FLASH (version 1.2.11) [40], short reads were removed using command “split_libraries_fastq.py” in QIIME (version 1.9.1) [41] and did dereplication using Vsearch (version 2.15.2) [42]. Following the above steps, a dataset of 2,174,676,759 nucleotides in 4,837,655 high-quality merged reads was compiled. Then, based on 97% pairwise identity by QIIME’s open reference Operational Taxonomic Units (OTU) picking strategy, sequences were clustered into OTUs using UCLUST [43]. It produced 3,511,624 counts and 229,331 OTUs using the Greengenes 16S rRNA database as a reference (13-8 release). Taxonomic classification of the representative sequence and alignment for each OTU was performed to the Ribosomal Database Project’s classifier using PyNAST [44] embedded in QIIME using default parameters. Next, a phylogenetic tree was generated from the alignment file by FastTree in QIIME. Chimeric OTUs were filtered using Vsearch software and all OTUs identified as belonging to chloroplast and mitochondria were removed from the data set using the command “filter_taxa_from_otu_table.py” in QIIME. To reduce low-abundance and spurious OTUs, those with at least a 0.01% total abundance in the OTU table were kept.

### Statistical analysis

For alpha-diversity analyses, all samples were rarefied to 3617 reads per sample to consider for differences in the number of reads across samples. We estimated the Shannon diversity (H´) and observed OTU richness indices using the package Phyloseq in R [45]. Statistical analysis for alpha diversity was done with the function “Kruskal.test” or “pairwise.Wilcox.test” in the R base. For beta-diversity analyses, OTU tables were normalized by the variance stabilizing transformation (VST) method using the package DESeq2 [46] in R. Bray-Curtis distance was calculated from the normalized OTU tables using the “ordinate” function of the R package Vegan [47]. PCOA and CAP analysis using the Bray-Curtis distance was calculated using the “plot_ordination” function from the R package Phyloseq. Permutational multivariate analysis of variance (PERMANOVA) was determined with the function “adonis” in the R package Vegan using the Bray-Curtis dissimilarities and a maximum of 500 permutations. The information of beta and alpha diversity considering all factors and their interactions were listed in Supplementary Table 5 and Supplementary Table 6, respectively. The core microbiome determined in 70% of the samples of each species with a relative abundance threshold value above 0.01%, was identified using command “compute_core_microbiome.py” in Qiime using the vegetative and reproductive datasets separately and then Venn diagrams were created using VennPainter [48]. Data transformation was used expressed as log-transformed counts, followed by DESeq2 which was used to evaluate differentially abundant taxa. Data visualization was performed with the R package ggplot2 [49]. KEGG ortholog pathway from PICRUSt [50] was used to predict the functional capabilities of rhizobacterial communities using an OTU table created through a closed-reference picking workflow. STAMP [51] was then used to analyze statistical differences between two groups of samples using FDR and produce Fig. 3.

## Results

In this study, we obtained 3,511,624 total high-quality 16S rRNA sequence reads, which clustered into 229,331 OTUs at 3% distance sequence dissimilarity. After discarding uncharacterized, low-abundance, and spurious OTUs, 1124 OTUs were identified.

### The rhizosphere is influenced by the plant developmental stage in wheat

**Fig. 2:**
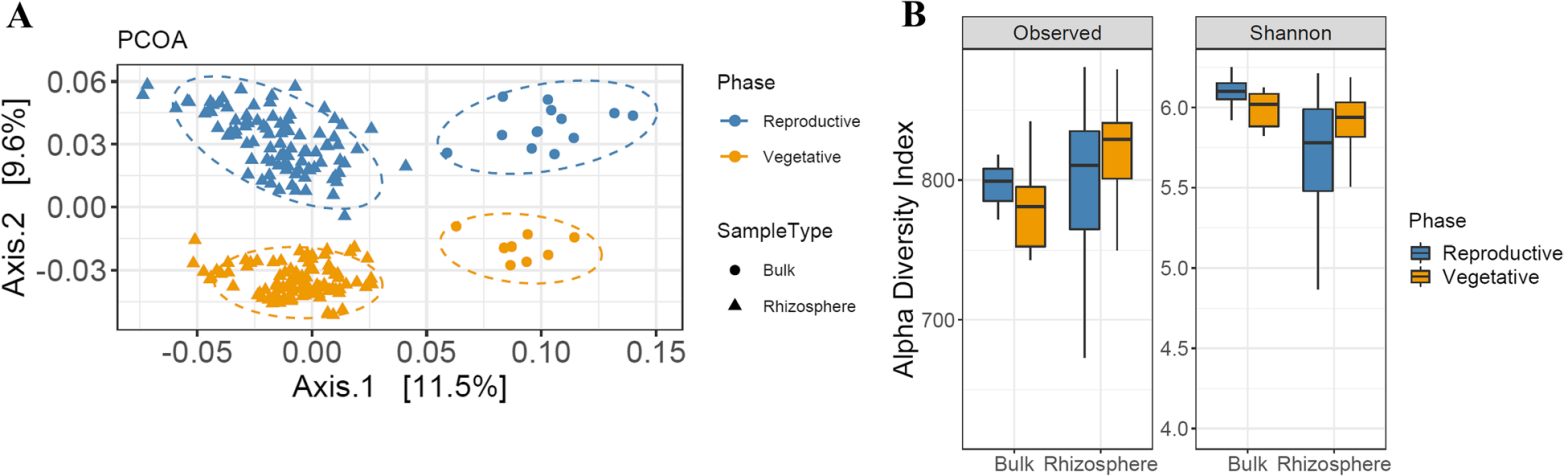
Root-associated microbial communities are separable by sample type and developmental stage. **A** PCoA using the Bray-Curtis metric indicates that the largest separation between bacterial communities is sample type (PCo 1) and the second-largest source of variation in the developmental stage (PCo 2). **B** alpha-diversity measurements between different sample types and developmental stages indicate a decreasing gradient in microbial diversity of the rhizosphere from vegetative to reproductive

At first, we visualized the Bray-Curtis distances between samples using principal coordinates analysis (PCoA) to investigate the dissimilarity between sample types (rhizosphere vs bulk soil) across two plant growth stages (vegetative vs reproductive). Generally, we observed a clear separation between the microbiome of two sample types and different time points (Fig. 2A). PCOA analysis declared that the sample type explained 11.5% of the variance and the growth stage could explain 9.6% of the overall variance of the data (Fig. 2A). PERMANOVA based on distance matrices (Adonis) also displayed a significant contribution of the sample type (8.9%, *P* = 0.001; Bray-Curtis; Supplementary Table 5), as well as the developmental stage (8.6%, *P* = 0.001, Bray-Curtis; Supplementary Table 5) and sample type-by-developmental stage interaction (19.2%, *P* = 0.001; Bray-Curtis; Supplementary Table 5). These data clearly showed that the bacterial community found in the wheat rhizosphere was distinct from bulk soil in both vegetative and reproductive stages. Furthermore, PCOA analyses generated using OTU phylogenetic relationships with both weighted UniFrac distance matrix, sensitive to OTU relative abundances, and unweighted UniFrac distance which is sensitive to unique taxa confirmed the observed discrimination of rhizobacterial communities at two developmental stages, although a substantial reduction of the variance was exhibited based on unweighted UniFrac distance (16.4%) when compared to the weighted (28.3%) (Supplementary Fig. 1).

We also determined the diversity of the sequencing data and total OTU richness for different sample types and two developmental stages (Fig. 2B). Generally, alpha diversity indices were significantly different in the rhizosphere and bulk soil (*P* = 3.213e-06 and *P* = 0.004 for Shannon diversity and observed OTU richness, respectively; Pairwise Wilcoxon test; Supplementary Table 6). Similarly, there were statistically significant differences between two developmental time points concerning overall community properties (*P* = 0.003042 and *P* = 0.002741 for Shannon diversity and observed OTU richness, respectively; Pairwise Wilcoxon test; Supplementary Table 6). We considered that vegetative rhizobacterial communities had a larger diversity and richness when compared to the rhizosphere at the reproductive time (*P* = 1.907e-05 and *P* = 0.0006 respectively; Pairwise Wilcoxon test; Supplementary Table 6), suggesting that specific bacteria may be changing through developmental stages.

**Fig. 3:**
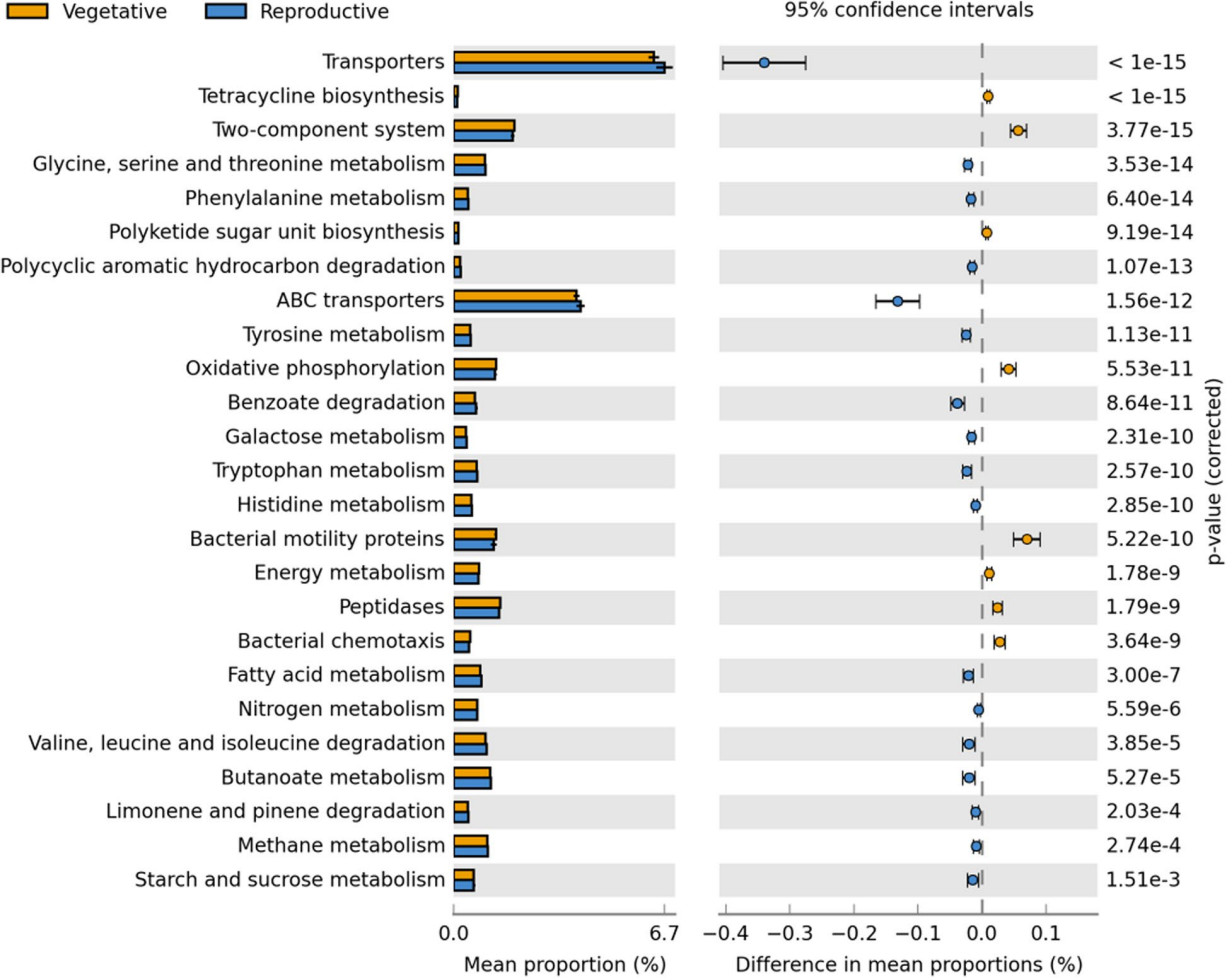
Extended error bars showing statistical differences of 16S rRNA gene-predicted functional profiles obtained with “PICRUSt” revealed that there is some variation in the datasets of two developmental stages. Corrected *p*-values were calculated using FDR correction (*p* < 0.05)

The functional profile of KEGG ortholog pathways obtained with PICRUSt showed that most of the functions associated with degradation of organic acids, amino acids, fatty acids, and more complex polycyclic aromatic hydrocarbons such as limonene and pinene along with ABC transporters were higher during the reproductive time (Fig. 3). However, young seedlings showed a higher abundance of predicted functions related to energy metabolism and oxidative phosphorylation, production of antimicrobial compounds such as Tetracycline, peptidase activity, bacterial motility, signal transduction (two-component system), and chemotaxis. Overall, it might suggest a variation in the functional datasets of two developmental stages.

Further investigation exhibited OTUs with the significant differences between sample types mostly belonged to the families *Cyanobacteria*, *OD1*, *Acidobacteria*, *Planctomycetes*, and *Gemmatimonadetes*, and those OTUs could explain most of the observed variation among plant developmental stages was mainly part of the phyla *Bacteroidetes*, *Firmicutes*, *Verrucomicrobia*, *Fibrobacteres* and *Actinobacteria* (Supplementary Fig. 2). Detailed information of the top 2o significant families has also been mentioned in Supplementary Fig. 2. Altogether, these data indicated that the wheat rhizosphere was colonized by taxonomically distinct communities compared to bulk soils and specific rhizobacterial taxa, as well as bacterial pathways, were probably influenced by plant development.

### Rhizobacterial communities associated with wheat species changed during evolution

**Fig. 4:**
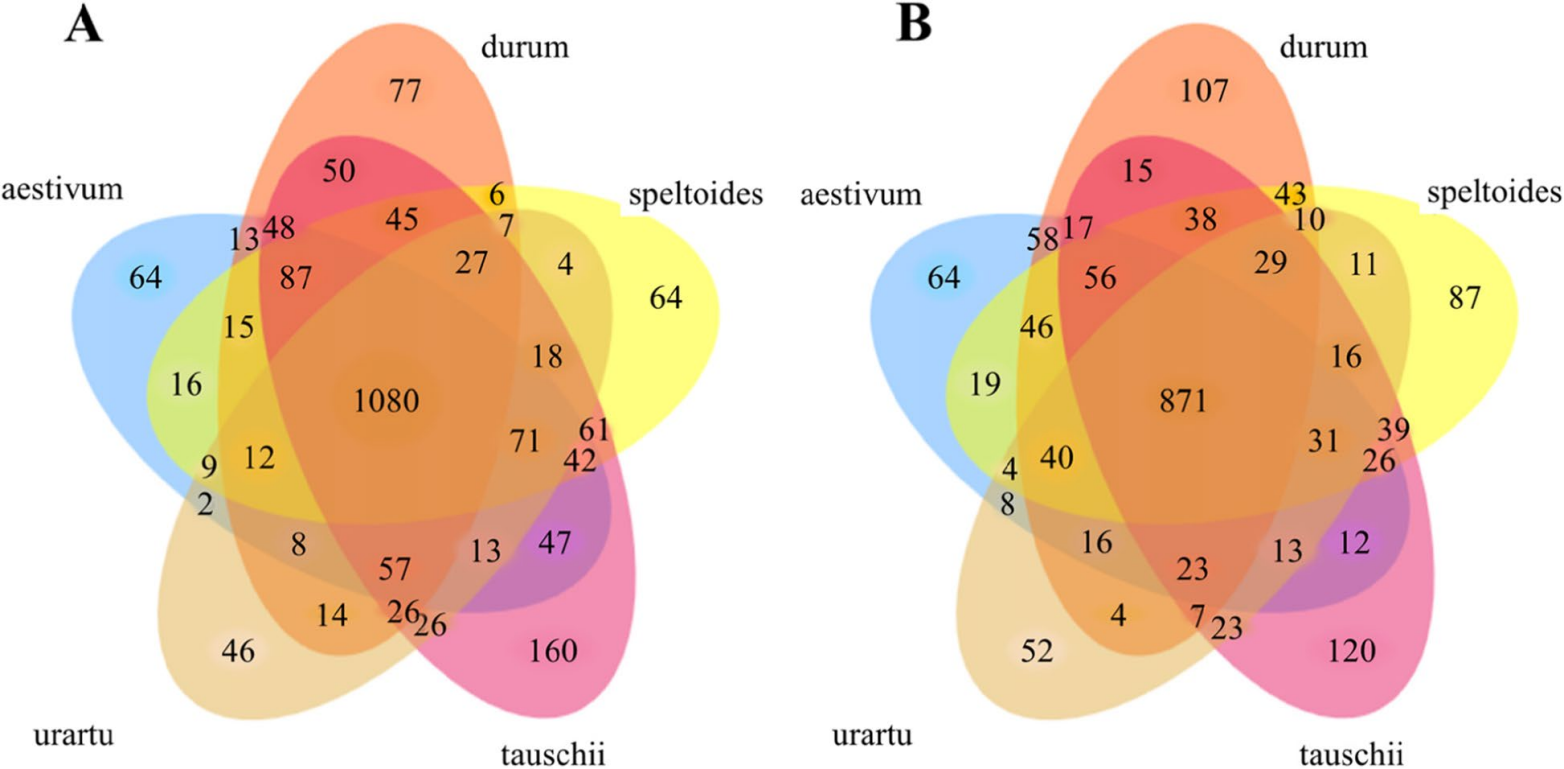
Core microbiome analysis between different wheat species at two developmental stages shows differences in shared OTUs. This diagram reveals the overlapping OTUs between plant species based on OTU presence or absence in 70% of the samples for each corresponding plant species. The OTU table filtered to remove OTUs with < 0.01% abundance and normalized for sequence depth was used to calculate the core microbiome. **A** Vegetative stage. **B** Reproductive time

The majority of OTUs discovered in this analysis were common in the rhizosphere of all six wheat species across two different developmental stages (Fig. 4A, B). Similarly, the large number of OTUs associated with the rhizosphere of the wild species (*T. urartu*, *Ae. speltoides*, and *Ae. tauschii*) was also present in the domesticated wheat plants (*T. durum* and *T. aestivum*), although there were differences in shared OTUs between different plant species and distinct developmental times. In both domesticated species *T. aestivum* and *T. durum*, we observed a larger fraction of bacterial OTUs shared with the wild species *Ae. tauschii* at the vegetative time (39.9 and 38.17% for *T. aestivum* and *T. durum*, respectively) and with *Ae. speltoides* at the reproductive stage (29.38 and 30.45% for *T. aestivum* and *T. durum*). The percentage of shared OTUs between two domesticated species and *T. urartu* was also the least at both vegetative and reproductive stages (33 and 27% at vegetative and reproductive stages, respectively for both *T. aestivum* and *T. durum*) (Fig. 4A, B).

**Fig. 5:**
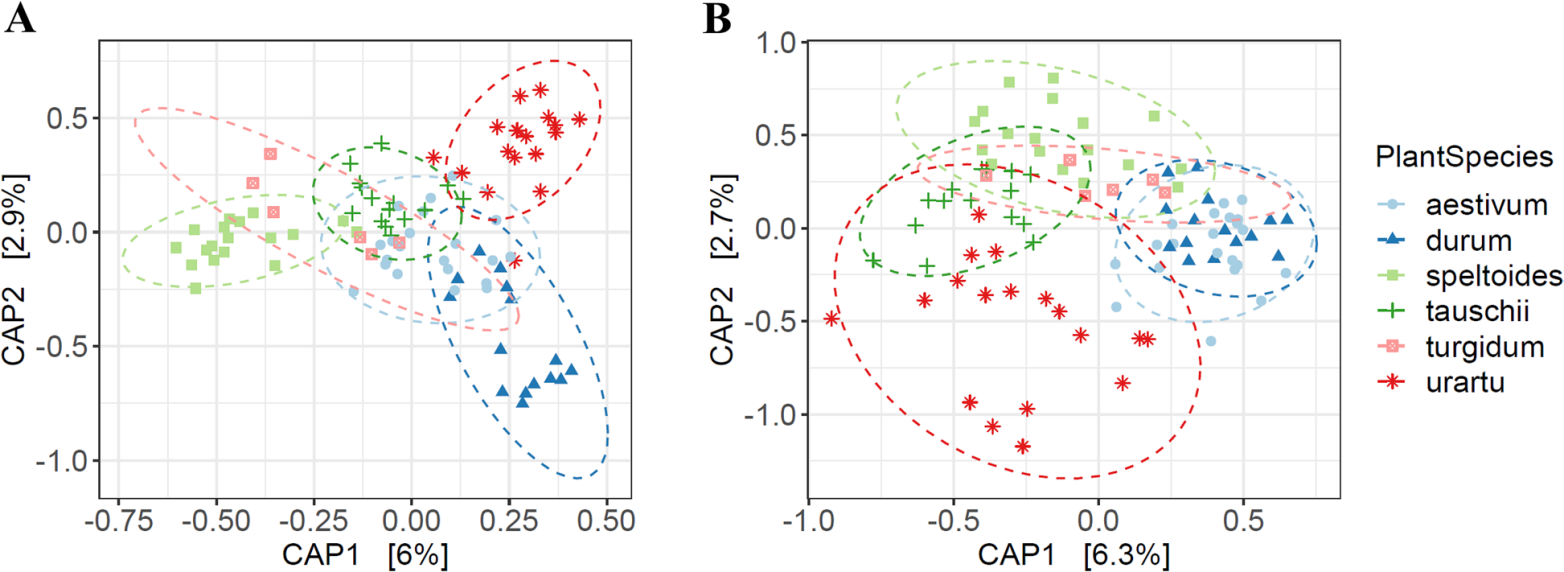
Host species significantly affect bacterial communities in the rhizosphere. Ordination of CAP analysis using the Bray-Curtis metric constrained to wheat species. **A** Vegetative stage. **B** Reproductive stage

**Fig. 6:**
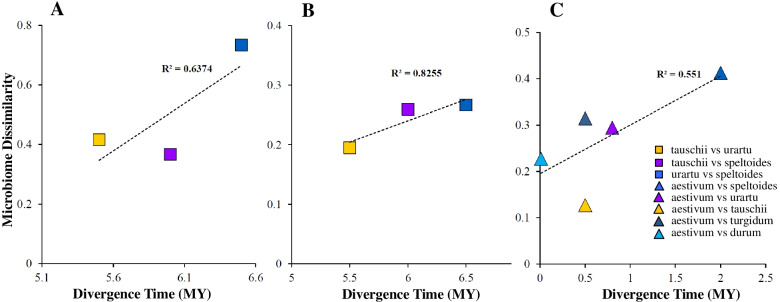
Microbiome dissimilarity as a function of host divergence shows that closely related wheat species have more similar rhizobacterial communities. We performed a Pairwise comparison of host phylogenetic distance and dissimilarity distance between the associated rhizobacterial communities of wild diploid species at (**A**) Vegetative stage, (**B**) Reproductive stage, and those of (**C**) *T. aestivum* at the vegetative stage

We separated the whole dataset to focus on each developmental stage individually and conducted a CAP analysis using the Bray-Curtis distance, where both vegetative and reproductive data sets displayed clustering by plant species (Fig. 5A, B). Concerning the rhizobacterial communities at the vegetative stage, the CAP plots revealed the rhizobacterial communities of two wild species *Ae. speltoides* and *T. urartu* exhibited the highest variation between species, while samples of *Ae. tauschii* clustered more closely together in the middle of speltoides and urartu arrangements (Fig. 5A). In the rhizobacterial communities at the reproductive stage, the clusters mainly correlated to the different domestication history of wheat (wild species vs cultivated), starting from the wild species (urartu, tauschii, speltoides, turgidum) at one end and the domesticated species (durum and aestivum) at another end (Fig. 5B). Our analysis also revealed a positive and linear relation between rhizobacterial communities of three wild diploid species (speltoides, urartu, and tauschii) in both vegetative and reproductive stages as compared the value of their divergence time according to data from Marcussen and coworkers with the pairwise community composition dissimilarity [33] (Fig. 6A, B). CAP results also confirmed that the samples were derived from two wild species *T. turgidum* (genome AABB) and *Ae. speltoides* (genome BB) generally clustered close to each other at both vegetative and reproductive stages, while *T. durum* (another species for genome AABB) exhibited a clear clustering far from those of *T. turgidum* and *Ae. speltoides* as depicted in Fig. 5A and B (Fig. 5A, B). In the case of tauschii species, the CAP plot (Fig. 5A, B) and pairwise community composition dissimilarity analysis (Fig. 6A, B) showed that there was a distinct clustering for genome DD between samples from speltoides and urartu in both vegetative and reproductive times, however, rhizobacterial communities of these three wild species accounted for a greater variance at vegetative than reproductive stage (CAP1 at vegetative vs CAP2 at the reproductive stage) (Fig. 5A, B). Similarly, aestivum formed a visible group between the tauschii and durum clusters at the vegetative stage, whereas it grouped closer to the durum at the reproductive time and exhibited a distinct arrangement far from that of genome DD (Fig. 5A, B). Moreover, Bray-Curtis dissimilarity between the rhizosphere microbiome of each pair of *T. aestivum* and its relatives was linearly correlated with the divergence time between the species at the vegetative stage (Fig. 6C), but not at the reproductive time. Strangely, the rhizobacterial communities of *T. durum* were more different from those of each wild relative than predicted based on the evolutionary time. Since there was a large dissimilarity especially between durum and two wild species *T. turgidum* (R = 0.65, *P* = 0.001) and *Ae. speltoides* (R = 0.727, *P* = 0.001), indicating that divergence time alone cannot define wheat rhizosphere microbiome diversification, and domestication may influence the rhizobacterial community of durum even in the early growth stage. While both vegetative and reproductive data sets were probably influenced by domestication (wild vs cultivated), the influence of this factor was greater in the reproductive than in the vegetative communities (5.8% vs 3.2% of the variation, *P* = 0.001, and *P* = 0.001 respectively) (Fig. 5A, B). Together, these results exhibited that bacterial communities of wheat species were perhaps affected by evolutionary relationships such as divergence time and domestication across different developmental stages.

The pattern of alpha diversity measurements showed that although the levels of microbial diversity differed significantly across different species (*P* = 2.911e-07; Kruskal-Wallis; Supplementary Table 6), domestication did not generally predict alpha diversity measurements (*P* = 0.8261; Pairwise Wilcoxon test; Supplementary Table 6), suggesting that rarer taxa undervalued in Shannon diversity measurements, may be more sensitive to domestication than are common taxa.

**Fig. 7:**
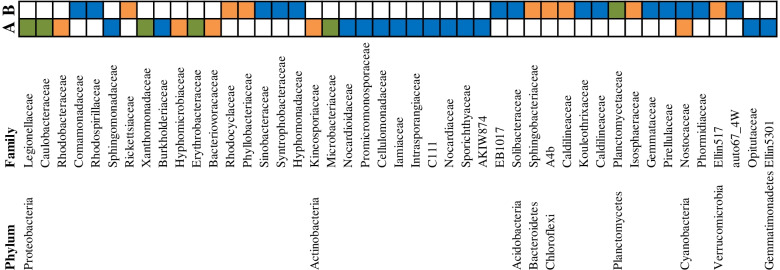
differentially family-level relative abundances and their respective phyla exhibit differences during domestication. Differential families in (**A**) domesticated and (**B**) wild wheat species during different developmental stages were presented with different colors. The colors orange, blue, and green relate to differentially taxa at vegetative, reproductive, and common in both developmental times, respectively

As mentioned above, rhizobacterial communities of wild and domesticated species accounted for a greater variance at the reproductive than the vegetative stage. Although the changes in rhizobacterial OTU abundance caused by domestication were not as strong as those caused by individual plant species, several families belonging to *Actinobacteria* including *Promicromonosporaceae*, *Nocardioidaceae*, *Microbacteriaceae*, *Sporichthyaceae*, *Lamiaceae*, *Intrasporangiaceae*, *Cellulomonadaceae*, *Nocardiaceae,* and *C111* as well as families *Xanthomonadaceae*, *Erythrobacteraceae*, *Legionellaceae*, *Burkholderiaceae,* and *Caulobacteraceae* belonging to *Proteobacteria* revealed significant increases in domesticated crops at the reproductive time when compared to wild species (Fig. 7). On the other hand, various bacterial taxa included *Sphingobacteriaceae*, *Rhodocyclaceae*, *Phyllobacteriaceae*, *Comamonadaceae*, *Planctomycetaceae*, *Solibacteraceae*, *Hyphomonadaceae*, *Rhodospirillaceae*, *Rickettsiaceae*, *Syntrophobacteraceae*, and *Caldilineaceae* along with two families *Nostocaceae* and *Phormidiaceae* belonging to *Cyanobacteria* were also differentially more abundant in wild species (Fig. 7). Altogether, these results exhibited that wheat rhizobacterial communities were influenced not only by the plant species but also by the factor domestication and the interaction of this factor with the plant development stage, which explained together 14.1% of the variance in the wheat rhizosphere microbiome (Supplementary Table 5).

### Host genetic affects the rhizosphere microbiome of wheat

To investigate the relationship between wheat genotypes and the rhizosphere microbiome, different wheat genotypes spanning six species within the genus *Triticum* and *Aegilops* were sampled. PERMANOVA indicated that rhizobacterial communities of wheat genotypes accounted for 24.2% of the overall variation between microbial communities (*P* = 0.001; Bray-Curtis; Supplementary Table 5). Moreover, the interaction of host genotype with plant developmental stage explained the largest fraction of overall variation (42 to 52%, Bray-Curtis; Supplementary Table 5). Through CAP analyses generated using the Bray-Curtis distance matrix, we surveyed genetic effects for genotypes within individual wheat species (Supplementary Fig. 3; A-F). CAP described genetic differences as having the second-largest source of variation behind the developmental stage. Based on CAP analysis, it was also declared that wheat genotypes within a given wheat species showed a small but significant fraction (5–8% of variation) of the total variation in beta-diversity. (Supplementary Fig. 3; A-F).

**Fig. 8:**
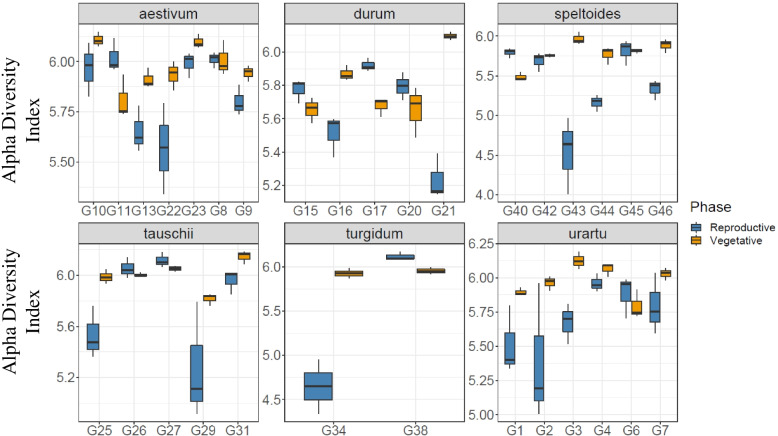
Alpha diversity measurement within the rhizosphere displayed significant differences between the genotypes. Estimated Shannon (H′) index in the bacterial communities associated with each genotype for the six wheat species across two developmental stages, shown with ± SE

Similarly, alpha diversity measurements displayed significant differences between the rhizosphere microbiome of genotypes (*P* = 9.554e-08 and *P* = 1.277e-05; Kruskal-Wallis, for Shannon diversity and observed OTUs respectively; Supplementary Table 6) and genotype-by developmental stage interactions (*P* = 6.953e-011 and *P* = 5.7197e-09; Kruskal-Wallis, for Shannon diversity and observed OTUs respectively; Supplementary Table 6). Across all 31 genotypes studied, two tauschii genotypes, G31 and G27, had the rhizosphere with the highest diversity at vegetative and reproductive stages respectively, however, two genotypes G40 and G43 from *Ae. speltoides* showed a lower diversity at vegetative and reproductive stages (Fig. 8). Considering different genotypes within individual plant species we found that genotypes in *Ae. tauschii*, *T. aestivum,* and *T. turgidum* exhibited significant Shannon diversity (*P* = 0.004, *P* = 0.0028, *P* = 0.044 respectively; Kruskal-Wallis; Supplementary Table 6), whereas, in other plant species, host genotype had little influence over community measurements of Shannon diversity (*P* = 0.94, *P* = 0.58, *P* = 0.15; Kruskal-Wallis, respectively for durum, speltoides, and urartu; Supplementary Table 6).

To explain which OTUs accounted for the genotypic effects in each rhizosphere, we performed differential OTU abundance analyses between the genotypes across two developmental stages separately. In total, we found 215 OTUs that were influenced by the plant genotype in at least one developmental stage. Respectively 54, 164, and 92 differential OTUs were specific for vegetative, reproductive, and common in both developmental stages. The reproductive time had the most OTUs that were significantly impacted by genotype. Moreover, our differential family-level abundance analysis identified bacteria that appear selectively favored in genotypes of each wheat species across different developmental stages (Supplementary Fig. 4).

### Wheat bacterial communities differ significantly among the rhizosphere of modern cultivars and landraces

**Fig. 9:**
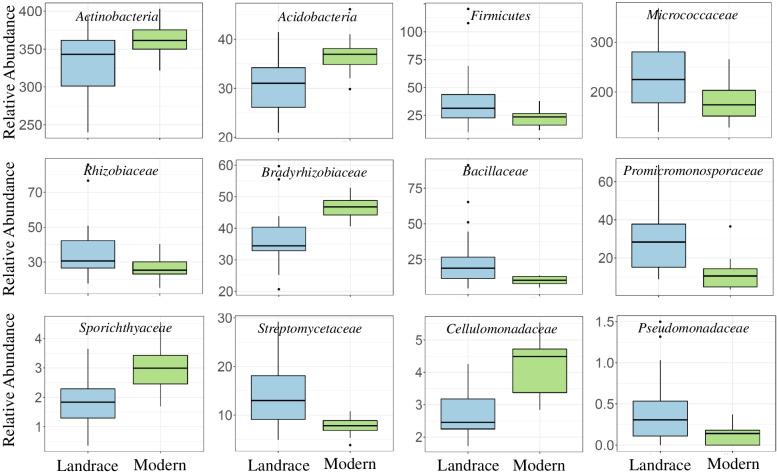
Bacterial community composition differed significantly among rhizosphere samples of modern cultivars and landraces at reproductive time. The relative abundance (%) of the phyla and families from wheat breeding groups including landrace and modern cultivars at the reproductive time was used

We separately evaluated the impact of classical breeding in the rhizosphere microbiome of modern and landrace genotypes. Across all domesticated samples (durum and aestivum), bacterial community structure differed significantly among modern cultivars and landraces (R2 = 2%, *P* = 0.047; Bray-Curtis; Supplementary Table 5). Furthermore, the reproductive time had a greater breeding effect on the wheat microbiome than vegetative stage (R2 = 7.5%, *P* = 0.001 and R2 = 4.5%, *P* = 0.001 respectively; Bray-Curtis). CAP results also confirmed that there was a small variation (2%) in the datasets of modern and landraces in the rhizosphere bacterial communities (Supplementary Fig. 5). Considering alpha diversity measurements, although the bacterial communities of the modern cultivars and landraces displayed equivalent degrees of OTU richness (*P* = 0.096; Observed OTUs, Supplementary Table 6), bacterial communities of landraces were significantly less diverse than those of modern plants (*P* = 0.037, Shannon diversity; Supplementary Table 6). We also conducted a differential representation of phyla and families between modern cultivars and landraces with samples taken from the reproductive stage (Fig. 9). It revealed several bacterial members from phyla *Actinobacteria*, *Proteobacteria,* and *Firmicutes* displaying significant variation in their relative abundance depending on the wheat breeding group (modern vs landrace). Our result showed an intensive depletion in the members of *Bacillaceae*, *Planococcaceae*, *Rhizobiaceae*, *Promicromonosporaceae*, *Actinosynnemataceae*, *Micrococcaceae*, *Micromonosporaceae*, *Pseudomonadaceae*, and *Streptomycetaceae* observed in the rhizosphere of modern cultivars than landraces (Fig. 9). On the other hand, modern cultivars tended to have high relative abundances of *Acidobacteria* as well as the members of bacterial families *Rubrobacteraceae*, *Cellulomonadaceae*, and *Sporichthyaceae* from *Actinobacteria* and *Bradyrhizobiaceae* from *Proteobacteria* (Fig. 9). Together, these results revealed that local wheat landraces probably interact with rhizosphere microbial communities in different ways from those of modern cultivars at a reproductive time.

## Discussion

Our study determined the bacterial community in the rhizosphere of different wheat species as compared with bulk soil during the vegetative and reproductive stages. These data showed that the bacterial community found in the wheat rhizosphere was distinct from bulk soil in both vegetative and reproductive stages, exemplifying the well-characterized rhizosphere effect [11, 17]. This result is expected since the rhizosphere environment influences the bacterial community via root exudation and nutrient input [7, 38].

In addition, the root-associated microbial community structure is affected by the plant growth stage. Variations in the composition of root-associated microbiomes during plant development have been revealed in several previous studies [6, 8, 52], and were suggested to be caused by changes in root exudation [37]. Although the compositions and quantities of the root exudates were not assessed in this study, the functional profile from PICRUSt showed that there was some variation in the datasets of two developmental stages. Most of the functions enriched during the vegetative stage were associated with energy metabolism, peptidase activity, signal transduction, biosynthesis of antibiotics, and chemotaxis, suggesting a higher metabolic activity of bacterial cells in a competitive environment, as various species of microorganisms coexist and compete for diffusing nutrients. However, predicted degradation pathways along with ABC transporters were higher during the reproductive stage. ABC transporters are involved in the translocation of a wide range of substrates such as sugars, ions, and complex organic molecules from the cells [53], suggesting the changes in ABC transporter may have a connection with the changes in the abundance and composition of root exudates. Since the bacterial community may be shifted during plant development from its capacity to exploit amino acids to the utilization of carbohydrates as the plant matured. However, future investigations should compare this result through metagenomics with a focus on function.

In recent years, numerous investigations have hypothesized that the composition of the microbiome may be shaped by the evolutionary history of eukaryotic hosts [13]. This suggests that the more phylogenetically distant the eukaryotic host, the more distinct their rhizobacterial communities [10]. A strong relationship between phylogenetic distance and rhizosphere microbiome dissimilarity has been reported in the literature for *Poaceae* species [10], *Brassica napus* genotypes [11], different species from monocots and dicots [12], but not for distant relatives of Arabidopsis [13]. Our study revealed a positive linear correlation between phylogenetic distance and rhizosphere bacterial community dissimilarity in wheat wild ancestors at both vegetative and reproductive stages. Likewise, Bray-Curtis dissimilarity between the rhizosphere microbiome of each pair of *T. aestivum* and their relatives at the vegetative stage was linearly correlated with the divergence time between the species, but not for *T. durum*. One possible explanation is that species *T. durum* was influenced by a faster rate of the shift during the vegetative stage since the rhizosphere microbiome of durum was more different from those of each wild relative than predicted based on the evolutionary time. Thereby, we theorize that divergence time alone cannot probably define wheat rhizosphere microbiome diversification, and domestication may influence the root-associated microbiome of durum even in the early growth stage. Recently, a small but significant fraction of the total variance in the structure of the bacterial communities at the vegetative stage of durum wheat was correlated to domestication [36]. In addition, our finding advocated that by hybridization between *T. durum* and *Ae. tauschii*, rhizobacterial communities changed in the rhizosphere of *T. aestivum* at the vegetative stage*.* Therefore, we observe that the rhizosphere communities of the *T. aestivum* have greater similarity to wild species compared to durum. Previous studies identified the D genome of *Ae. tauschii* as a determinant of mycorrhiza colonization of its progeny hexaploid wheat lines [54]. We also suggest the genomic content of *Ae. tauschii* probably plays a major role in the bacterial interactions at the root-soil interface of *T. aestivum* at the vegetative stage.

Considering rhizobacterial communities at the reproductive stage, we presented that the rhizosphere communities of cultivated species aestivum and durum and also wild species *T. turgidum* were more similar to *Ae. speltoides* than other wild diploid relatives when comparing the value of their pairwise community composition dissimilarity. However, two cultivated species durum and aestivum exhibited the smallest between-species variation at the reproductive time that was independent of ploidy, suggesting that the microbiome derived from an ancient plant species *Ae. speltoides* have been probably influenced by domestication-related processes at the reproductive time. A possible explanation for this result may be the differences between wheat species for root system development [35], photosynthetic activity-related traits [55], nutrient uptake efficiency, and the response to nutritional limitations [56], inoculation with mycorrhizal fungi [57], and other plant-related factors. Recent studies have demonstrated that phenotypic distance between plant host [21, 35], root exudates [38], and specific genetic pathways [58, 59] can have significant effects on microbiome composition and diversity. Moreover, modern wheat has been bred for traits that yielded the largest aboveground production such as plant height, spike size, heading date/growth duration, grain size, and other domestication-related attributes. It has been speculated that these processes have also influenced belowground traits including root architecture [34, 35] and root exudate composition [37, 38], which in turn may modify rhizosphere microbial communities. Similarly, distinct rhizosphere communities were explained for domesticated plants compared with their respective wild ancestors [18, 19, 36]. In addition, Ito et al. [60] have recently reported that the A genome carried by *T. urartu* has a dominant effect on the rhizospheric bacterial community structure of Triticum species over the B and D genomes. They observed a difference between Triticum and Aegilops groups, but not within Triticum species in terms of rhizobacterial communities. Comparatively, we showed that the genome contents of *Ae. tauschii*, *Ae. speltoides* and domestication-related changes are probably the significant factors determining the composition of root-associated bacterial communities in cultivated wheat. In addition, our results revealed 33 and 27% shared OTUs between *T. urartu* and cultivated species at the vegetative and reproductive stages, respectively; suggesting a major effect of *T. urartu* might also exist. Therefore, to understand the wheat root-associated microbiome, its polygenetic mosaic nature needs to be taken into consideration. Further studies with a larger number of genotypes may provide a deeper understanding of the actual diversity amongst these species.

In the current study, the enrichment of members of the families *Actinobacteria* and *Proteobacteria* were detected in domesticated crops. By contrast, several phyla such as *Verrucomicrobia*, *Cyanobacteria*, and *Bacteroidetes* represented a much larger fraction of the communities of wild species. Comparatively, in *Phaseolus vulgaris*, going from wild to cultivated, a gradual increase in the relative abundance of *Actinobacteria* and *Proteobacteria* and a decrease in the relative abundance of *Bacteroidetes* have been reported [21]. *Bacteroidetes* have also been reported with the higher relative abundance in the rhizosphere of wild plant species and wild crop relatives, including *Beta vulgaris* subsp. *maritima* [61], *Cardamine hirsuta* [13], *Lactuca serriola* [62] and *Hordeum vulgare* subsp. *spontaneum* [17]. It has been suggested that *Bacteroidetes* are capable of digesting polysaccharides in wild plants and promoting plant growth under poor environmental conditions [63]. In addition, *Chitinophagaceae* and *Sphingobacteriaceae,* which belong to the *Bacteroidetes*, have been proposed for their potential role in protection against soil-borne pathogens [64]. Although the influence of the observed changes in microbiome composition on the growth and health of wheat will be the subject of future studies, wild relatives of wheat may provide a new perspective into plant genes associated with microbiome assembly.

Wheat genetic background also provided a large significant amount of variation. This result may be explained by the fact that these genotypes represent different species originating from various geographical regions; however, genotypes within every individual species demonstrated a minor but significant difference in beta diversity. Similar findings have also been reported by former studies [22, 23]. It is interesting to note that there is a fine-scale regulation of the rhizobacterial communities by wheat genotypes, as a total of 215 differentially abundant OTUs were genotype-specific and the strongest genetic effects on microbes were observed at the reproductive stage when 164 OTUs were genotype-specific. We also observed a strong genotypic variation within species of *T. aestivum* and *Ae. tauschii*. This evidence suggests an extensive and selective control by wheat genotypes surveyed on associated rhizobacterial communities. Since these controls are genetically based, they might show potential breeding targets if the associated bacteria are revealed to be positively related to growth, health, or other positive traits in subsequent studies.

Concerning the effect of wheat breeding programs, it was found that modern breeding practices might have had a significant impact on microbiome-plant interactions, especially at the reproductive stage. This is in agreement with evidence on barley, durum, and bread wheat [17, 29, 36]. For instance, Spor et al. [36] showed the depletion of nitrifiers in the modern wheat cultivars compared to the primitive domesticated landraces, suggesting that modern breeding programs decreased the coupling between plant and rhizosphere microbes that are potentially important for plant nutrient availability. We also observed a stronger selection of bacterial taxa in primitive landraces than modern genotypes that may improve plant nutrient uptake. For example, several differentially abundant bacteria such as *Rhizobiaceae*, *Micrococcaceae*, and *Streptomycetaceae,* which are much enriched in the rhizosphere of landraces comparing modern cultivar, are associated with the diazotrophic bacterial community, with potential benefits in the N-fixation process. Moreover, several lineages from families *Pseudomonadaceae*, *Bacillaceae*, *Rhizobiaceae*, *Burkholderiaceae*, and *Microccocaceae* show more differential abundance in the rhizosphere of landraces comparing modern counterparts are accepted to have P-solubilizing activity [65]. In addition, the current study showed that the diversity of the microbial community in modern cultivars was significantly larger than that of primitive landraces based on the Shannon index. In agreement with the present result, a previous study demonstrated that the semi-dwarf wheat genotypes, as a consequence of the Green Revolution and modern breeding, displayed larger diversity comparing tall plants [29]. Since extending overall microbial activity or niche saturation may be important to competitive exclusion of pathogens [66], increasing proportional diversity found in modern genotypes probably supports improved pathogen suppressiveness. Based on these observations, we advocate that modern breeding for increasing yield and disease resistance combined with the transition from low-input soils to high-input agricultural soil changed the interactions between plants and their microbiomes, with potential consequences for nutrient cycling and competition with soil-borne pathogens in the rhizosphere. Further surveys are needed to determine the impact of these enriched microbes on the growth and health of wheat.

## Conclusions

This study performed with different genotypes of Triticum and Aegilops species is the first study showing that the genome contents of ancestral species *Ae. speltoides*, and *Ae. tauschii* can be a significant factor determining the composition of root-associated bacterial communities in domesticated bread wheat. It is also indirect evidence that shows a very extensive range of host traits and genes are probably involved in host-microbe interactions and understanding the wheat root-associated microbiome need to take into consideration of its multilevel polygenetic mosaic nature. Although it was found that domestication and modern breeding practices might have had a significant impact on microbiome-plant interactions, we observed an extensive and selective control by wheat genotypes on associated rhizobacterial communities especially within primitive landraces of *T. aestivum* and accessions of *Ae. tauschii*. Since these controls are genetically based, they might show potential breeding targets if the root-associated bacteria are revealed to be positively related to growth, health, or other positive traits in subsequent studies.

## Supplementary Information

**Additional file 1: Supplementary Figure 1.** Unconstrained PCoA reveals distinct clustering of microbiomes of different developmental stages (A) Unconstrained PCoA using the Bray-Curtis distance metric. (B) Unconstrained PCoA using the weighted UniFrac distance metric. (C) Unconstrained PCoA using the unweighted UniFrac distance metric. **Supplementary Figure 2.** The wheat rhizosphere microbiome was colonized by distinct taxa. (A) and (C) Correspond to rhizosphere vs bulk soil comparison in phylum and family level respectively, and (B) and (D) Correspond to the rhizosphere of vegetative stage vs. that of reproductive time respectively in phylum and family level. To find the most important up-regulated features among the differentially expressed bacterial taxa, the MicrobiomeSeq package was used to detect respectively the top 10 and 20 differentially abundant Phyla and families among different comparison groups, and mean decrease accuracy values of differentially abundant taxa were calculated. **Supplementary Figure 3.** Host signature reveals a second distinct clustering of the variation in the rhizosphere microbiomes of individual wheat species after accounting for the variation present among developmental stages. Ordination of CAP analysis using the Bray-Curtis metric constrained in genotypes of (A) *T. aestivum*, (B) *T. durum*, (C) *T. turgidum*, (D) *T. urartu*, (E) *Ae. tauschii*, (F) *Ae. speltoides* across two developmental stages. **Supplementary Figure 4.** Differentially family-level relative abundance exhibits differences between genotypes of six wheat species across two developmental stages. Differential families in genotypes of (A) *T. aestivum*, (B) *T. durum*, (C) *T. turgidum*, (D) *T. urartu*, (E) *Ae. tauschii*, (F) *Ae. speltoides,* during different developmental stages, were represented with different colors. The colors orange, blue, and green show differential families at vegetative, reproductive, and both sampling time points respectively. **Supplementary Figure 5.** Bacterial community structure differs significantly among rhizosphere samples of modern cultivars and landraces. Ordination of CAP analysis using the Bray-Curtis metric constrained to factor *Breeding*. **Supplementary Table 1.** Field and grassland soils used as a seed bank of the microbiome in this study. **Supplementary Table 2.** Soil chemical and physical analysis. **Supplementary Table 3.** Wild wheat accessions used in this study. **Supplementary Table 4.** Modern and Landrace varieties used in this study. **Supplementary Table 5.** PERMANOVA of the bacterial communities associated with wheat plants considering all factors and their interactions. Numbers in sub-indices indicate the degrees of freedom and residuals of each F test. **Supplementary Table 6.** Experimental factors predicting alpha-diversity of bacterial communities associated with the rhizosphere of wheat. Statistical support was done with the function “kruskall.test” or “pairwise.Wilcox.test” in the R base considering all factors and their interactions. All *P* values were corrected for multiple comparisons using the FDR correction. Numbers in sub-indices indicate the degrees of freedom and residuals of each F test.

## Data Availability

The datasets generated for this study can be accessed from the European Nucleotide Archive under accession number PRJEB46375.

## Abbreviations

OTU: Operational taxonomic units
PCR: Polymerase chain reaction
PCoA: Principal coordinates analysis
CAP: Canonical analysis of principal coordinates
VST: Variance stabilizing transformation
PERMANOVA: Permutational multivariate analysis of variance
FDR: False discovery rate
KEGG: Kyoto encyclopedia of genes and genomes
