# Supplementary material for: Changes in root microbiome during wheat evolution
Source: BMC Microbiol. 2022 Feb 26;22:64. doi: 10.1186/s12866-022-02467-4 (PMC8881823; doi:10.1186/s12866-022-02467-4)
Supplement: Supplementary file 1 — Additional file 1: Supplementary Figure 1. Unconstrained PCoA reveals distinct clustering of microbiomes of different developmental stages (A) Unconstrained PCoA using the Bray-Curtis distance metric. (B) Unconstrained PCoA using the weighted UniFrac distance metric. (C) Unconstrained PCoA using the unweighted UniFrac distance metric. Supplementary Figure 2. The wheat rhizosphere microbiome was colonized by distinct taxa. (A) and (C) Correspond to rhizosphere vs bulk soil comparison in phylum and family level respectively, and (B) and (D) Correspond to the rhizosphere of vegetative stage vs. that of reproductive time respectively in phylum and family level. To find the most important up-regulated features among the differentially expressed bacterial taxa, the MicrobiomeSeq package was used to detect respectively the top 10 and 20 differentially abundant Phyla and families among different comparison groups, and mean decrease accuracy values of differentially abundant taxa were calculated. Supplementary Figure 3. Host signature reveals a second distinct clustering of the variation in the rhizosphere microbiomes of individual wheat species after accounting for the variation present among developmental stages. Ordination of CAP analysis using the Bray-Curtis metric constrained in genotypes of (A) T. aestivum, (B) T. durum, (C) T. turgidum, (D) T. urartu, (E) Ae. tauschii, (F) Ae. speltoides across two developmental stages. Supplementary Figure 4. Differentially family-level relative abundance exhibits differences between genotypes of six wheat species across two developmental stages. Differential families in genotypes of (A) T. aestivum, (B) T. durum, (C) T. turgidum, (D) T. urartu, (E) Ae. tauschii, (F) Ae. speltoides, during different developmental stages, were represented with different colors. The colors orange, blue, and green show differential families at vegetative, reproductive, and both sampling time points respectively. Supplementary Figure 5. Bacterial commun [file 12866_2022_2467_MOESM1_ESM.docx]

**SUPPLEMENTARY MATERIAL**

**Supplementary figures**

**Supplementary Figure 1** Unconstrained PCoA reveals distinct clustering of microbiomes of different developmental stages (**A**) Unconstrained PCoA using the Bray-Curtis distance metric. (**B)** Unconstrained PCoA using the weighted UniFrac distance metric. (**C**) Unconstrained PCoA using the unweighted UniFrac distance metric.

**Supplementary Figure 2** The wheat rhizosphere microbiome was colonized by distinct taxa. (**A**) and (**C**) Correspond to rhizosphere vs bulk soil comparison in phylum and family level respectively, and (**B**) and (**D**) Correspond to the rhizosphere of vegetative stage vs. that of reproductive time respectively in phylum and family level. To find the most important up-regulated features among the differentially expressed bacterial taxa, the MicrobiomeSeq package was used to detect respectively the top 10 and 20 differentially abundant Phyla and families among different comparison groups, and mean decrease accuracy values of differentially abundant taxa were calculated.

**Supplementary Figure 3** Host signature reveals a second distinct clustering of the variation in the rhizosphere microbiomes of individual wheat species after accounting for the variation present among developmental stages. Ordination of CAP analysis using the Bray-Curtis metric constrained in genotypes of (**A**) *T. aestivum*, (**B**) *T. durum*, (**C**) *T. turgidum*, (**D**) *T. urartu*, (**E**) *Ae. tauschii*, (**F**) *Ae. speltoides* across two developmental stages.


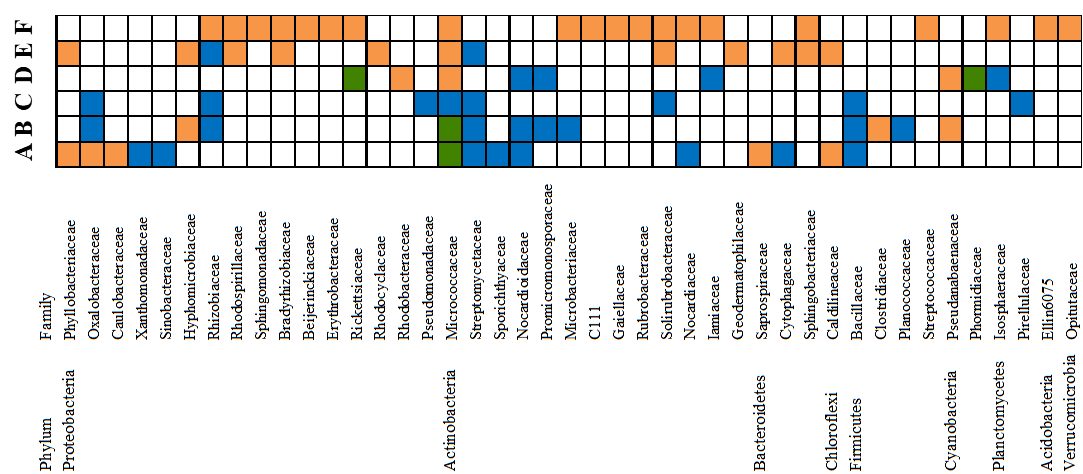


**Supplementary Figure 4** Differentially family-level relative abundance exhibits differences between genotypes of six wheat species across two developmental stages. Differential families in genotypes of (**A**) *T. aestivum*, (**B**) *T. durum*, (**C**) *T. turgidum*, (**D**) *T. urartu*, (**E**) *Ae. tauschii*, (**F**) *Ae. speltoides,* during different developmental stages, were represented with different colors. The colors orange, blue, and green show differential families at vegetative, reproductive, and both sampling time points respectively.

**Supplementary Figure 5** Bacterial community structure differs significantly among rhizosphere samples of modern cultivars and landraces. Ordination of CAP analysis using the Bray-Curtis metric constrained to factor *Breeding*.

**Supplementary Tables**

**Supplementary Table 1** Field and grassland soils used as a seed bank of the microbiome in this study

| Soil | Origin | Region | Latitude | Longitude | Altitude  (m above sea level) |
| --- | --- | --- | --- | --- | --- |
| Soil_1  Soil_2  Soil_3  Soil_4  Soil_5  Soil_6 | grassland  grassland  grassland  grassland  field  field | Iran  Iran  Iran  Iran  Iran  Iran | N 36° 37ʹ 14ʹʹ  N 37° 41ʹ 08ʹʹ  N 31° 51ʹ 50ʹʹ  N 36° 43ʹ 06ʹʹ  N 39° 07ʹ 01ʹʹ  N 34° 19ʹ 49ʹʹ | E 53° 36ʹ 39ʹʹ  E 48° 16ʹ 32ʹʹ  E 49° 40ʹ 48ʹʹ  E 49° 43ʹ 26ʹʹ  E 44° 46ʹ 15ʹʹ  E 47° 17ʹ 48ʹʹ | 1054  1293  659  1921  1491  1352 |

**Supplementary Table 2** Soil chemical and physical analysis

| Soil Parameters | | Amount |
| --- | --- | --- |
| Carbon and Nitrogen | NH4 (mg/kg)  NO3 (mg/kg)  Total C (%)  Total N (%) | 10.5  7  3.10  0.23 |
| Total minerals | P (mg/kg)  K (mg/kg)  Na (mg/kg)  Ca (mg/kg)  Mg (mg/kg) | 6.52  571.5  80.84  23.2  3.6 |
| Physical Analysis | Clay (%)  Silt (%)  Sand (%)  Texture | 17  22  61  Sandy Loam |
| EC (ds/m) | | 1.5 |
| PH | | 6.5 |

**Supplementary Table 3** Wild wheat accessions used in this study

| Code | Genus | Species | Genome | Region | Latitude | Longitude | Altitude  (m above sea level) |
| --- | --- | --- | --- | --- | --- | --- | --- |
| G1  G2  G3  G4  G6  G7  G34  G38  G25  G26  G27  G29  G31  G40  G42  G43  G44  G45  G46 | Triticum  Triticum  Triticum  Triticum  Triticum  Triticum  Triticum  Triticum  Aegilops  Aegilops  Aegilops  Aegilops  Aegilops  Aegilops  Aegilops  Aegilops  Aegilops  Aegilops  Aegilops | Urartu  urartu  urartu  urartu  urartu  urartu  turgidum  turgidum  tauschii  tauschii  tauschii  tauschii  tauschii  speltoides  speltoides  speltoides  speltoides  speltoides  speltoides | AA  AA  AA  AA  AA  AA  AABB  AABB  DD  DD  DD  DD  DD  BB  BB  BB  BB  BB  BB | Iran  Iran  Iran  Iran  Iran  Iran  Iran  Iran  Iran  Iran  Iran  Iran  Iran  Iran  Iran  Iran  Iran  Iran  Iran | N 33° 53ʹ 30ʹʹ  N 34° 27 22  N 33° 31ʹ 28ʹʹ  N 36° 25ʹ 26ʹʹ  N 34° 47ʹ 32ʹʹ  N 36° 17ʹ 33ʹʹ  N 39° 22ʹ 41ʹʹ  N 47° 55ʹ 03ʹʹ  N 38° 19ʹ 9ʹʹ  N 38° 27ʹ 42ʹʹ  N 37° 06ʹ 47ʹʹ  N 37° 12ʹ 39ʹʹ  N 37° 28ʹ 16ʹʹ  N 33° 29ʹ 34ʹʹ  N 34° 50ʹ 08ʹʹ  N 35° 02ʹ 45ʹʹ  N 34° 50ʹ 39ʹʹ  N 33° 25ʹ 03ʹʹ  N 37° 21ʹ 16ʹʹ | E 50° 51ʹ 29ʹʹ  E 46° 16ʹ 18ʹʹ  E 47° 22ʹ 25ʹʹ  E 47° 25ʹ 22ʹʹ  E 51° 11ʹ 34ʹʹ  E 47° 85ʹ 21ʹʹ  E 44° 05ʹ 43ʹʹ  E 39° 37ʹ 42ʹʹ  E 46° 25ʹ 28ʹʹ  E 45° 29ʹ 22ʹʹ  E 50° 06ʹ 53ʹʹ  E 49° 59ʹ 23ʹʹ  E 49° 18ʹ 39ʹʹ  E 46° 44ʹ 30ʹʹ  E 46° 30ʹ 58ʹʹ  E 46° 21ʹ 44ʹʹ  E 47° 41ʹ 48ʹʹ  E 46° 37ʹ 43ʹʹ  E 57° 14ʹ 52ʹʹ | 1673  1509  1961  1551  1796  1472  2198  53  1406  1516  46  1  -22  1684  1602  1574  2067  1617  1309 |

**Supplementary Table 4** Modern and Landrace varieties used in this study

| Code | Genus | Species | Genome | Region | Name | Type |
| --- | --- | --- | --- | --- | --- | --- |
| G8  G9  G10  G11  G13  G22  G23  G15  G16  G17  G20  G21 | Triticum  Triticum  Triticum  Triticum  Triticum  Triticum  Triticum  Triticum  Triticum  Triticum  Triticum  Triticum | aestivum  aestivum  aestivum  aestivum  aestivum  aestivum  aestivum  durum  durum  durum  durum  durum | AABBDD  AABBDD  AABBDD  AABBDD  AABBDD  AABBDD  AABBDD  AABB  AABB  AABB  AABB  AABB | Iran  Iran  Iran  Iran  Iran  Iran  Iran  Iran  Iran  Iran  Iran  Iran | Alvand  1-32-4382  Sorkh-Tokhm  Sabalan  Sari-Bughda  1-27-6275  Kavir  Karkheh  Zardak  Gerdish  Kooleh  Chehel-Daneh | Modern  Landrace  Landrace  Modern  Landrace  Landrace  Modern  Modern  Landrace  Landrace  Landrace  Landrace |

**Supplementary Table 5** PERMANOVA of the bacterial communities associated with wheat plants considering all factors and their interactions. Numbers in sub-indices indicate the degrees of freedom and residuals of each F test.

| Dataset | Factor | F | R2 | P |
| --- | --- | --- | --- | --- |
| Total | Sample type _1,205_  Growth Stage _1,205_  Sample type:Stage _3,203_ | 20.115  19.35  16.165 | 0.08935  0.08625  0.19283 | 0.001 ***  0.001 ***  0.001 *** |
| Data_Rhizosphere | PlantSpecies _5,180_  PlantSpecies:Growth Stage _11,174_  Genotype _30,155_  Genotype:Growth Stage _61,124_  Domestication _1,184_  Domestication:Growth Stage _3,182_ | 3.0012  4.5367  1.653  1.7182  4.6623  9.9678 | 0.07695  0.22288  0.24239  0.19314  0.02471  0.14112 | 0.001 ***  0.001 ***  0.001 ***  0.001 ***  0.001 ***  0.001 *** |
| Data_Rhizosphere_vegetative | Plantspecies _5,87_  Genotype _30,62_  Domestication _1,91_ | 2.93  2.0563  3.0493 | 0.14412  0.49874  0.03242 | 0.001 ***  0.001 ***  0.001 *** |
| Data_Rhizosphere_reproductive | Plantspecies _5,87_  Genotype _30,62_  Domestication _1,91_ | 2.6397  1.8605  5.6009 | 0.13172  0.47376  0.05798 | 0.001 ***  0.001 ***  0.001 *** |
| Data_Cultivated | Breeding _1,70_  Breeding:Growth Stage _3,68_ | 1.6447  1.5606 | 0.02006  0.17064 | 0.047 *  0.001 *** |
| Data_ Aestivum | Genotype _6,35_  Genotype:Growth Stage _13,28_ | 1.4232  2.3291 | 0.19613  0.51954 | 0.003 **  0.001 *** |
| Data_ Durum | Genotype _4,25_  Genotype:Growth Stage _9,20_ | 1.2014  2.1951 | 0.16123  0.49693 | 0.077  0.001 *** |
| Data_ Turgidum | Genotype _1,10_  Genotype:Growth Stage _3,8_ | 1.4542  1.9873 | 0.12695  0.42701 | 0.053  0.001*** |
| Data_Speltoides | Genotype _5,30_  Genotype:Growth Stage _11,24_ | 1.4438  2.2104 | 0.19396  0.50326 | 0.001 ***  0.001 *** |
| Data_Tauschii | Genotype _4,25_  Genotype:Growth Stage _9,20_ | 1.3733  2.2897 | 0.18014  0.50748 | 0.02 *  0.001 *** |
| Data_ Urartu | Genotype _5,30_  Genotype:Growth Stage _11,24_ | 1.297  2.0424 | 0.17774  0.48349 | 0.018 *  0.001 *** |

**Supplementary Table 6** Experimental factors predicting alpha-diversity of bacterial communities associated with the rhizosphere of wheat. Statistical support was done with the function "kruskall.test" or "pairwise.Wilcox.test" in the R base considering all factors and their interactions. All P values were corrected for multiple comparisons using the FDR correction. Numbers in sub-indices indicate the degrees of freedom and residuals of each F test.

| Dataset | Factor | Shannon | | Observed | |
| --- | --- | --- | --- | --- | --- |
|  |  | chi-squared | P | Chi-squared | p |
| Total | SampleType _1,204_  Growth Stage _1,204_  SampleType:Growth Stage _3, 202_ | 21.685  8.7821  39.572 | 3.213e-06  0.003042  1.313e-08 | 8.2837  8.972  18.28 | 0.004  0.002741  0.000385 |
| Data_Rhizosphere | Growth Stage _1,183_  Plantspecies _5,179_  Plantspecies:Growth Stage _11,173_  Genotype _30,154_  Genotype:Growth Stage _61,123_  Domestication _1,183_  Domestication:Growth Stage _3,181_ | 18.28  38.561  59.318  88.924  160.48  0.048244  19.202 | 1.907e-05  2.911e-07  1.241e-08  9.554e-08  6.953e-11  0.8261  0.0002484 | 11.738  23.955  42.805  74.258  146.32  0.22789  14.846 | 0.0006123  0.0002215  1.174e-05  1.277e-05  5.719e-09  0.6331  0.001953 |
| Data_Rhizosphere_vegetative | Plantspecies _5,87_  Genotype _30,62_  Domestication _1,91_ | 29.217  82.417  0.25482 | 2.103e-05  8.825e-07  0.6137 | 31.591  77.097  4.2729 | 7.159e-06  5.122e-06  0.03872 |
| Data_Rhizosphere_reproductive | Plantspecies _5,86_  Genotype _30,61_  Domestication _1,90_ | 16.963  76.138  1.421 | 0.00457  6.986e-06  0.2332 | 8.8065  71.631  0.71944 | 0.117  2.926e-05  0.3963 |
| Data_Cultivated | Breeding _1,70_  Breeding:Growth Stage _3,68_ | 4.3202  14.182 | 0.03766  0.002668 | 2.7582  10.195 | 0.09676  0.01698 |
| Data_Aestivum | Genotype _6,35_  Genotype:Growth Stage _13,28_ | 19.942  32.043 | 0.002836  0.002367 | 22.881  29.762 | 0.0008374  0.005095 |
| Data_Durum | Genotype _4,25_  Genotype:Growth Stage _9,20_ | 0.73978  26.041 | 0.9463  0.002012 | 3.3223  26.927 | 0.5054  0.001438 |
| Data_Turgidum | Genotype _1,9_  Genotype:Growth Stage _3,7_ | 4.0333  8.197 | 0.04461  0.04211 | 2.7  5.2879 | 0.1003  0.1519 |
| Data_Speltoides | Genotype _5,30_  Genotype:Growth Stage _11,24_ | 3.7778  29.889 | 0.5818  0.00165 | 6.8356  26.303 | 0.2332  0.005849 |
| Data_Tauschii | Genotype _4,25_  Genotype:Growth Stage _9,20_ | 15.325  24.51 | 0.004073  0.003564 | 11.379  20.913 | 0.02262  0.01304 |
| Data_Urartu | Genotype _1,34_  Genotype:Growth Stage _11,24_ | 8.1141  24.405 | 0.1501  0.01113 | 6.9468  23.177 | 0.2246  0.01669 |
